# Improvement of hemodynamic performance using novel helical flow vena cava filter design

**DOI:** 10.1038/srep40724

**Published:** 2017-01-23

**Authors:** Ying Chen, Peng Zhang, Xiaoyan Deng, Yubo Fan, Yubin Xing, Ning Xing

**Affiliations:** 1 Key Laboratory for Biomechanics and Mechanobiology of Ministry of Education, School of Biological Science and Medical Engineering, Beihang University, Beijing, 100191, China; 2 Department of Infection Management and Disease Control, The General Hospital of People’s Liberation Army, Beijing, 100853, China

## Abstract

We propose a vena cava filter in which helical flow is created in the filter’s working zone to minimize filter blockage by trapped clots and facilitate the lysis of trapped clots. To validate this new design, we compared five helical flow inducers with different thread pitches in terms of blood flow patterns in the filter. The vena cava was reconstructed based on computed tomography images. Both the numerical simulation and *in vitro* experiment revealed that the helical flow inducer can effectively create a helical flow in the vessel, thereby subduing the filter structure’s adverse disruption to blood flow, and increasing flow-induced shear stress in the filter center. In addition, the smaller thread pitch helical flow inducer reduced the oscillating shear index and relative residence time on the vessel wall. Moreover, we observed that the helical flow inducer in the vena cava could induce flow rotation both in clockwise and counterclockwise directions. In conclusion, the new design of the filter with the smaller thread pitch inducer is advantageous over the traditional filter in terms of improving local hemodynamics, which may reduce thrombosis build-up after deployment.

Pulmonary embolism (PE) is a common complication among patients in intensive care units. The main pathologic cause of PE is deep venous thrombosis (DVT). DVT and PE remain a leading global cause of morbidity and mortality. Inferior vena cava (IVC) filters have been used to prevent recurrent PE in patients who are unresponsive to anticoagulation therapy or in who anticoagulation is contraindicated^1^. Although VCFs have been widely used in clinics, they still may endure major failures due to the buildup of thrombus in the filters themselves after deployment^2^.

The swirling motion of blood flow in the aortic arch is a typical example of the “form follows function” principle in the vascular system. The swirling blood flow guarantees that the inner surface of the ascending aortic wall will become smooth and that atherosclerotic plaques will be less likely to form in the ascending aorta^3^,^4^. One such blood flow pattern in the heart and arterial system is called helical or swirling flow. Helical flow has been suggested to improve the performance of endovascular stents^5^,^6^.

Herein, based on the principle of helical flow, we propose a VCF in which helical flow is created in the filter’s working zone to minimize filter blockage by trapped clots and facilitate the lysis of trapped clots. By analyzing numerical simulations and conducting *in vitro* experiment, we compare five filter models with helical flow inducers with different thread pitches. For comparison, we also evaluated a cone-shaped filter without an inducer.

## Simulation Methods

### Geometric model and meshing

**Figure 1:**
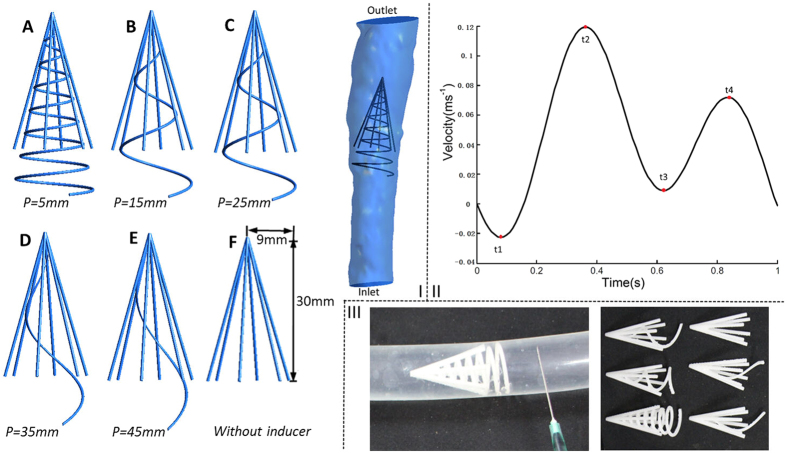
Models and Inlet waveform velocity. I: Diagram of five filters with five typical different thread pitches for helical flow inducers (**A,B,C,D,E**); Model F is a con-shaped filter of the same size without an inducer, and “*p*” represents the thread pitch of the inducer. The diagram also shows the filter in the vena cava used in the computations. II: Inlet IVC waveform velocity used in the pulsatile flow computations. III: Six filter models used *in vitro* experiment.

The VCF model consists of a helical flow inducer and a normal filter, which was created using the computer-aided design software Pro/Engineer (version Wildfire 4.0, Parametric Technology Corp, USA), based on the Greenfield filter^7^,^8^ and vena cava^9^ parameters from the literature. We simplified the normal filter as a cone-shaped stent and principally studied the helical flow inducer. Although the inclination angle was also a parameter of the helical flow inducer, to assemble the cone-shaped filter, the change in inclination angle was limited because of its cone form. Therefore, the most important parameter of the helical flow inducer was the thread pitch. As illustrated in Fig. 1(I), the filters with five helical flow inducers with different thread pitches (Cases A–E) were designed to be incorporated into the normal cone-shaped filter (F). The thread pitch of the helical flow inducers were 5, 15, 25, 35, and 45 mm, respectively. All of the helical flow inducers had the same inclination angle. The normal filter (F) was simplified to be composed of six symmetrical circular struts, and the diameter of each strut was 0.6 mm. The length of the inducer was 35 mm, and the entire filter length was 40 mm. The vena cava model was 109 mm in length, which was reconstructed based on the computed tomography images.

In each case, before the model was meshed, a Boolean operation of subtraction between the vena cava and filter model was performed. All the computational models were meshed with tetrahedral and hexahedral elements using ANSYS Meshing (ANSYS Inc., Canonsburg, PA). High density mesh elements were applied close to filters and vascular walls, in sizes of 0.1 mm and 0.3 mm respectively. The final volumes of the meshes were 3894763, 3880352, 3510841, 3387031, 3308648, and 3175689, respectively, for Case A to F.

### Numerical approaches

#### Assumptions

In this study, blood was assumed to be a homogeneous, incompressible non- Newtonian fluid^10^. The simulation was performed under laminar flow conditions^11^.

#### Governing equations

Flow simulations were performed based on the three-dimensional incompressible Navier–Stokes and continuity equations^12^:


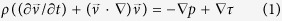



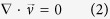


where 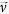 and p represent the fluid velocity vector and pressure, respectively. ρ = 1050 kg/m3 is the density of blood, andτis the tension tensor:


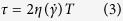


Here, T and 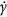 are the respective deformation tensor and shear rate, respectively, and *η* is the viscosity of blood that was a function of shear rate.

In present study, the Carreau model was used to calculate blood viscosity:


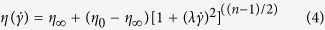


Where *η*_∞_ = 3.45×10^−3^ kg/(m s), *η*_0_ = 5.6×10^−2^ kg/(m s), n = 0.3568, and *λ* = 3.313 s^13^.

Here it should be mentioned that in arteries non-Newtonian and Newtonian flows yield essentially the same behavior, as suggested by Perktold K’s numerical simulation^10^. There are also many other non-Newtonian viscosity models such as, Canson, Oldroyd-B and Herschel-Bulkley *et al*. However, in the vena system, it is yet unknown how the adoption of different non-Newtonian model could influence simulation results.

#### Statistical analysis

To characterize the helical flow induced by the six filter models, the helicity density H_d_ (the kinetic helicity per unit volume) was calculated. H_d_, used to denote the swirl strength of a flow, is defined by Equation 5^14^. Equation 6 describes the area-weighted average of the helicity density^14^,^15^. Helicity plays a significant role in flow status and affects the evolution and stability of both turbulent and laminar flows^16^.


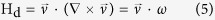



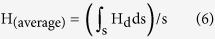


Here, 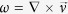 is the vorticity field of the flow, and *S* is the area of the cross-section.

The shear stress on the vessel wall throughout a cardiac cycle was evaluated using the time-averaged wall shear stress (TAWSS), defined as:


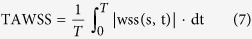


where *T* is a cardiac cycle period, WSS is the instantaneous wall shear stress vector, and s is the position on the vessel wall. To measure the directional change of WSS during the cardiac cycle, the oscillating shear index (OSI) was introduced. OSI denotes the changing frequency of the WSS direction, expressed as^17^:


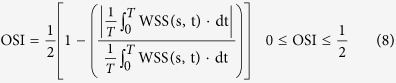


A zero value of OSI corresponds to unidirectional shear flow and when a purely oscillatory shear case happened, OSI value becomes to 1/2.

A new parameter termed relative residence time (RRT) was also calculated, defined as:


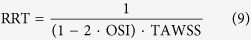


RRT reflects the residence time of flow particles near the wall, and also recommended as a single metric of low and oscillating shear stress^17^.

#### Boundary conditions and computation

In all cases, a steady flow simulation was performed first. This solution was then used as initial iteration data for further pulsatile flow simulations. For the steady flow simulation, a uniform inflow velocity profile with an axial velocity component of 0.1 m/s and a transverse velocity component equaling zero were used at the inlet. The pressure was set to 800 Pa^18^, whereas in the outlet cross-section, it was set to 0 Pa^19^. The vena cava vessel wall was assumed to be rigid and non-slippery.

In fact, the blood flow of the inferior vena cava (IVC) is affected by the contraction of the heart. The IVC has pulsatile waveforms with two peaks and reverse flow^10^ occurring on every cardiac cycle. We considered the Doppler blood flow waveforms of IVC reported by Bin Zhang^20^ and approximated them by the smooth periodic function plotted in Fig. 1(II). Therefore, for the pulsatile flow simulation, the time-dependent parabolic flow velocity waveform shown in Fig. 1(II) was set at the inlet, the other boundary conditions were set the same as the steady computation.

The finite volume method was adopted to solve the mass and momentum conservation equations using ANSYS Fluent CFD (ANSYS Inc., Canonsburg, PA). A pressure-based solver was used with a second-order upwind scheme for the momentum spatial discretization. The residual continuity and velocity were assigned the value of 1.0 × 10^–5^. Six cycles were required to obtain a convergence for the transient analysis, with 200 steps in each cycle (T = 1 s). The computational process spanned two weeks for each case.

## Simulation results

### Helicity

**Figure 2:**
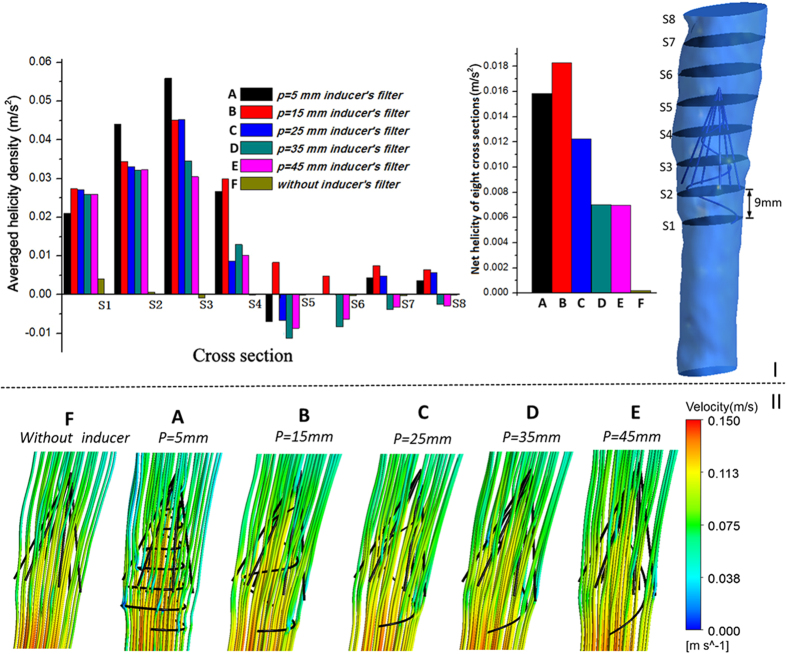
Helicity and streamline for the six cases. I: Eight cross-sections in the vena cava model, with each adjacent side distance measuring 9 mm; plots of area-weighted averages of H_d_ at eight representative cross-sections of six cases from steady flow computations; net helicity was also given. II: Peak systole (Time = t2) velocity streamline for the six cases from pulsatile flow computations.

To compare the average helicity in the different cases, we selected several representative cross-sections in the vena cava model for each case and plotted the area-weighted averages of helicity density H_d_ for eight representative cross-sections (S1–S8, Fig. 2I).

Based on the results of steady flow simulation, Fig. 2I shows that all the helical flow inducers, from Case A to E, induce the flow to non-zero helicity, whereas the helicity value of Case F reaches nearly zero on all sides. Some sides have negative helicity values, most notably Side 5. In fact, Equation (5) states that in flows with a rotational velocity field, when the velocity 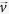 and vorticity *ω* vectors lie in the same direction, the helicity values are positive, as for Side 1 and Side2; when 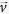 and *ω* lie in opposite directions, the helicity values are negative, as with Side 5 in Cases A, C, D, and E; helicity equals zero when the velocity and vorticity vectors lie in orthogonal directions (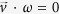), such as in Case F. In all the cases with helical flow inducers, a smaller thread pitch leads to higher net helicity values.

### Flow pattern

**Figure 3:**
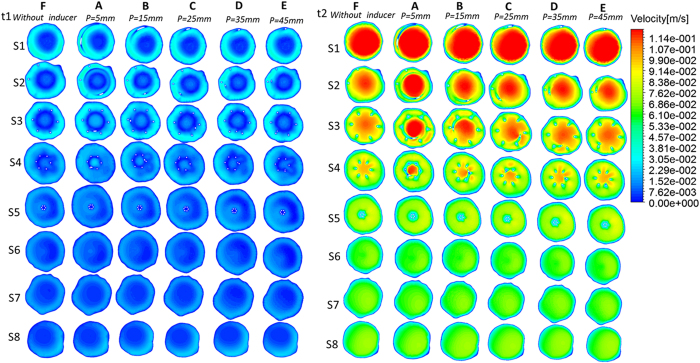
velocity distributions from pulsatile flow computations. Time = t1 and Time = t2 velocity distributions for each case.

**Figure 4:**
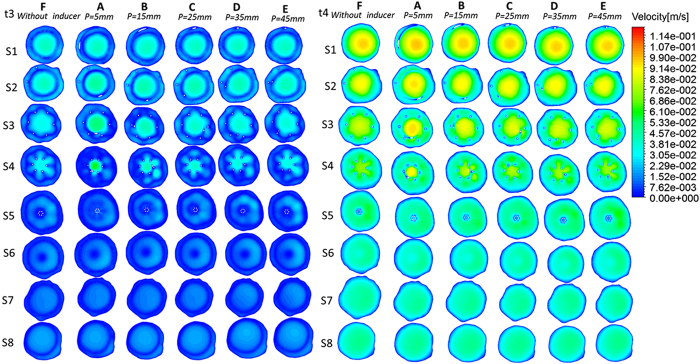
velocity distributions from pulsatile flow computations. Time = t3 and Time = t4 velocity distributions for each case.

Figures 3 and 4 present velocity profiles during the cardiac cycle within the eight planes of each vena cava. In general, except for the filter inside, each helical filter without significantly changed the velocity of blood flow within vena cava during the overall cardiac cycle among the different cases. However, the increase of the flow velocity in filter’s center is more pronounced after the placement of the helical flow inducer. For instance, at peak systole (Time = t2), as evident from the Fig. 3, the center velocity of Case A reaches as high as 0.12 m/s. From Case A to E, the area of high velocity inside the filter gradually decreases as the thread pitch of the helical flow inducer increases.

Figure 2II presents the peak systole (Time = t2) velocity streamlines in these six cases, colored by the velocity magnitude. The directions of the fluid elements inside these filters are locally induced by helical flow inducers. From Case A to F, the streamlines become increasingly smooth. Careful analysis reveals that in Cases A-E, the streamline curved in different directions, which may be related to the negative helicity value, discussed above.

### Time-averaged wall shear stress on the vessel wall and filter

**Figure 5:**
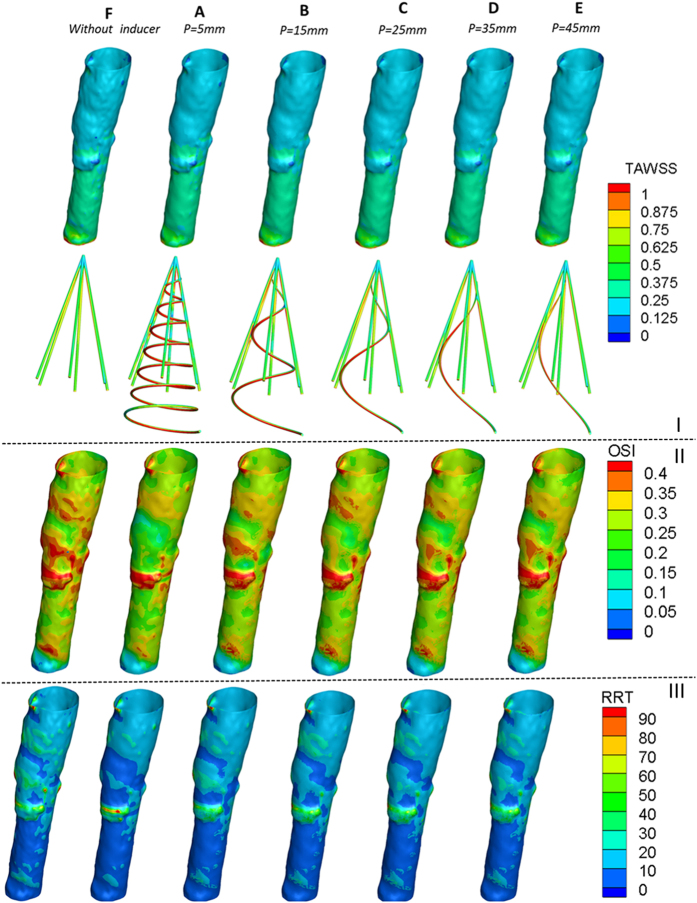
Contours of TAWSS, OSI and RRT from pulsatile flow computations. I: Contours of TAWSS on vena cava wall and filter. II: Contours of OSI on vena cava wall. III: Contours of RRT on vena cava wall.

The Time-averaged wall shear stress (TAWSS) contours of the vessel models after six types of filter deployments (A–F) are depicted in Fig. 5I. The TAWSS on the vessel wall was similar for all the cases. However, the TAWSS on the filter increased with the placement of the helical flow inducer. In particular, the areas with high TAWSS was gradually elevated as the thread pitch of the helical flow inducer decreased. Furthermore, Fig. 5I demonstrate that in each case, the TAWSS in the center was higher than at the outskirts. For instance, the Figure shows the discrepancy between the filter with the smallest thread pitch inducer and the filter without inducer. The highest value of TAWSS on the filter appeared for Case A (where TAWSS reached 1 Pa in the center), whereas the lowest value of TAWSS appeared in Cases F (where TAWSS reached only about 0.3 Pa).

### Oscillatory shear index and relative residence time

As observed in Fig. 5II, the OSI weakens after deployment of the helical flow inducer, which is more obvious in the vicinity of the inducer. This Figure also shows that areas of high OSI were easily observed in areas where the vessel’s diameter changed. Moreover, combined with the contours of the TAWSS (Fig. 5I), it is possible to see the region with high OSI values is usually located in the regions where the TAWSS is low.

Figure 5III also clearly demonstrates that the helical flow inducer decreases the RRT on the vessel wall, especially after the placement of a smaller thread pitch helical flow inducer. The effect of the inducer in reducing the RRT is most obvious near the helical flow inducer. Compared with the no-inducer model (F), the area-averaged mean RRT on the vessel wall is decreased from 20 to 10, especially for the smaller- thread-pitch helical model (A). Examining OSI and RRT in combination, it is possible to determine that low values of RRT almost correspond to low values of OSI and vice versa.

### *In vitro* experiment

#### Circulation perfusion system and filter model

For the *in vitro* experiment, a circulation perfusion system was set up. The six filter models tested in the study correspond to the numerical simulations above. The main difference was that the diameter of the filter strut was 1.6 mm, because struts that are too small are easily ruptured. All the models were made of a photosensitive resin using laser rapid prototyping technology. The total length of the filter was 40 mm, while that of the inducer was 35 mm. A flexible and transparent plastic hose with an inner diameter of 19 mm was used to simulate the vena cava. The plastic hose was fixed in the horizontal position and connected to a peristaltic flow pump and a flow meter. The flow rate through the test section could be varied by regulating the peristaltic flow pump, with the flow meter being used to measure the flow rate. The tested model was placed in the plastic hose, and a dye was injected upstream. In addition, a digital camera was used to record the flow state. A mixed fluid, consisting of 33.3% by volume of glycerol in water and having a density of 1.05 g/cm^3^ and a dynamic viscosity of 0.0033 Pa∙s, which closely approximates that of blood, was used for mimicking blood. This fluid was then maintained at room temperature. The velocity extent was 0.05–0.07 m/s in each case, which is approximately the flow rate used in the vitro experiment literature^21^. This is because the higher the velocity, the greater the difficulty in catching the flow streamline pictures. In order to ensure a laminar and fully developed flow entering the filter, it was ensured that the tested tube had a straight upstream length of more than a 140-mm. This corresponds to seven times the tube diameters and ensured a parabolic velocity profile under the flow conditions used in these experiments.

To further test the hemodynamic performance of the helical flow filter, in the *in vitro* experiment, some pig blood clots were added in the upstream for model A, mode F. In the first instance, a few blood clots (1.3 g, approximately 1.024 ml^3^ in volume) were released upstream; thereafter, in the subsequent instance more blood clots (5.5 g, approximately 4.332 ml^3^ in volume) were released.

## Experimental Results

**Figure 6:**
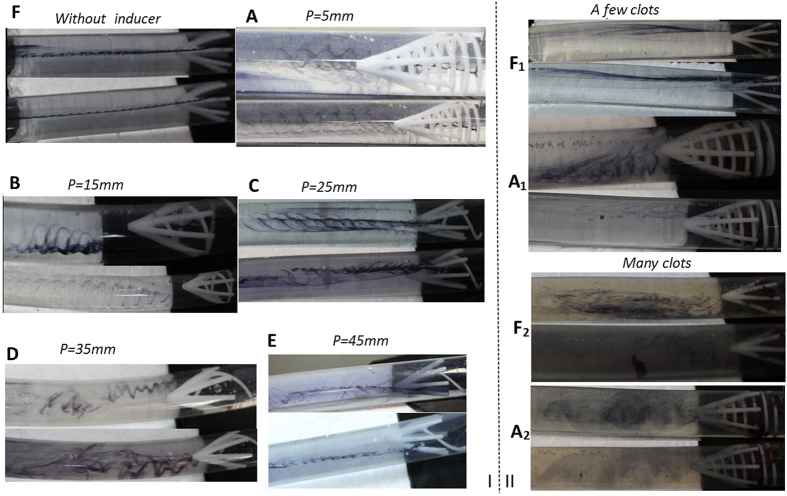
Experimental results. I: Velocity streamline for the six cases *in vitro* experiment. II: Velocity streamline with clots for case A, and F. A_1_, F_1_ represent a few clots; A_2_, F_2_ represent many clots.

Figure 6I illustrates the results for the six cases without blood clots. Compared to Case F without an inducer, the cases A-E with helical flow inducers induced more helical flow in the vena cava, particularly in the downstream of the filter. In general, for the Cases A-E, a filter with a smaller thread pitch inducer was prone to induce more helical flow. In other words, a smaller thread pitch helical flow inducer was not significantly worse than a filter with a larger thread pitch inducer. Whereas in Case F, the streamline was nearly straight, in Case A-E, evident eddies were observed in the downstream. Moreover, the helical flow eddies in the vena cava occurred in both clockwise and counterclockwise directions. The Specific flow rate for each case has been listed in Table 1.

As illustrated in Fig. 6II, in the case of blood clots, it can be observed that the umbrella or cone shape filter tends to trap clots in the center of the filter. This observation is consistent with previously reported results^21^. In case A, nearly all the clots were captured by the new novel filter; In case F, the filter can capture most of the clots. However, when the clots were increased from 1.3 g to 5.5 g, a few clots could not be captured by the filter in case F_2_. This contrasts with case A_2_ all the blood clots could be captured, even after being increased. Therefore, it could be claimed that the helical inducer itself also auxiliary captures the clots. As detailed in Table 1, it can be noted that for 1.3 g clots, the flow rate is 66 l/h for both A_1_ and F_1_. For 5.5 g clots, the flow rates for Case A_2_ and F_2_ decreased to 64 l/h and 63 l/h respectively. Whereas in Case A_2_, some eddies could be seen, in case F_2_, there were no evident eddies.

## Discussion and Conclusions

Filter restenosis is still a challenging problem even in modern medical era. An ideal VCF design should consider many factors, including geometry figure and hemodynamic feature after deployed in human. Many scholars, as Leask, Singer and Wang^22^,^23^,^24^ also suggest that hemodynamic considerations are an important factor in the filter designs. In present study, we proposed a VCF with a new concept in which helical flow is created in the vena cava to improve the local hemodynamics of the filter. To evaluate this design, numerical simulations and *in vitro* experiment were carried out to compare filters with helical flow inducers with various thread pitches under both steady and pulsatile flow conditions.

In Fig. 2I, we can see that all the helical flow inducers induce the flow to reach a non-zero helicity value, and sides 5–7 even have negative helicity values. To clarify the significance of a negative helicity value, we describe the helical flow; it is possible to define the basic quantity local normalized helicity (LNH) in Equation 10^25^:


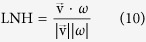


where 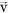 and *ω* represent, the fluid velocity vector and vorticity, respectively. The LNH is the local value of the cosine of the angle between the velocity and vorticity vectors. In the present study, when the helicity value is positive, the LNH is positive; when the helicity value is negative, the LNH is also negative. The sign of LNH is a useful indicator of the direction of rotation^25^,^26^. In Cases A, C, D, and E, some sides have negative helicity values and others have positive values, which indicate that the helical flow inducer in the filter could induce flow rotation in both clockwise and counterclockwise directions. Case B, only positive helicity values are observed, indicating that flow rotation is only induced in the clockwise directions. In Case F, the near-zero helicity value indicates that the flow is nearly without rotation.

Furthermore, our numerical simulation found that compared with the filter without inducer (Case F), the new design, particularly the filter with a smaller-thread-pitch inducer (Case A) not only induces helical flow but also partly reduces OSI and RRT in the filter vicinity (see Fig. 5). Hemodynamic studies have showed that high OSI and amplified relative residence time of blood flow would stimulate platelet aggregation and finally lead to thrombus formation^27^,^28^,^29^. Hoietal and Suhetal^30^,^31^ also indicated that high RRT is associated with the occurrence of atherosclerosis and stent thrombus. In addition, for the TAWSS, the filter with helical flow inducer is not significantly worse than the traditional one and higher TAWSS is observed at the center of the inducer. Low shear stress are usually associated with blood flow status and thrombosis^32^. Therefore, the hemodynamic performance of the new design may be better than the traditional design; in particular, a filter with a smaller-thread-pitch inducer might be able to prevent the development of thrombosis.

For the purpose of simplification, we used a normal filter comprising six symmetric wires, which is different from a clinical filter; Cone-shaped filters are very popular for use in clinics: for instance, Greenfield filters are typically cone-shaped. The main difference is the sinuous design of the filter legs, which may affect the blood flow state. Another limitation of the present study is that similar to most simulation studies of blood vessels, including arteries, we assumed the walls to be rigid, even though it is a well-known fact that the walls are elastic and made of spongy tissues. This assumption therefore does not deliver any information on the concentration profiles within the wall. However, compared with an arterial wall, the venous wall is much thinner, and the pressure is also very low. Therefore, we believe that these assumptions have a limited impact and that both the numerical simulation and *in vitro* experiment results remain significant.

In conclusion, this study results revealed that compared with the traditional filter, the new design, particularly the filter with smaller-thread-pitch helical flow inducer, not only induced helical flow but also could partly reduce the OSI and RRT. Therefore, the helical flow inducer could be used in VCF design. Intentionally inducing a helical flow in the vena cava vessel is one available method for solving the problems of acute DVT and PE. We believe that in vena cava the helical flow inducer could stabilize the blood flow, reduce the occurrence of the flow disturbance, reduce embolisms, and promote the lysis of trapped clots. However, the physiological roles of the helical flow filter were determined mostly from computational simulations and pilot *in vitro* experiments, which should be verified by *in vivo* experiments in a future study.

## Additional Information

**How to cite this article:** Chen, Y. *et al*. Improvement of hemodynamic performance using novel helical flow vena cava filter design. *Sci. Rep.* **7**, 40724; doi: 10.1038/srep40724 (2017).

**Publisher's note:** Springer Nature remains neutral with regard to jurisdictional claims in published maps and institutional affiliations.

## Figures and Tables

**Table 1:** Specific flow rate for each case.

case	A	B	C	D	E	F	A_1_	F_1_	A_2_	F_2_
Flow rate(l/h)	66	69	65	64	63	66	66	66	64	63

A_1_, F_1_ represent a few clots; A_2_, F_2_ represent many clots.
